# Supplementary material for: Traumatic brain injuries and problem gambling in youth: Evidence from a population-based study of secondary students in Ontario, Canada
Source: PLoS One. 2020 Oct 2;15(10):e0239661. doi: 10.1371/journal.pone.0239661 (PMC7531994; doi:10.1371/journal.pone.0239661)
Supplement: S1 Table — (DOCX) [file pone.0239661.s001.docx]

Table S1. Alternative logistic regression model of self-reported problem gambling by demographic characteristics, lifetime traumatic brain injuries, hazardous drinking and suicide attempts among secondary school students, OSDUHS 2011-2015, Ontario, Canada (*N*=9,198).

|  | Model 1 | | | |  | | Model 2 | | | | | | Model 3 | | | | | | |  |
| --- | --- | --- | --- | --- | --- | --- | --- | --- | --- | --- | --- | --- | --- | --- | --- | --- | --- | --- | --- | --- |
|  | AOR | 95% CI | | p | | AOR | | 95% CI | | | | p | | AOR | | 95% CI | | p |  |  |
| Male | 3.68 | 1.68 | 8.07 | *** | | 5.08 | | | 2.46 | 10.46 | *** | | 4.91 | | 2.34 | | 10.30 | *** | | |
| Grade | 1.16 | 0.95 | 1.42 | NS | | 0.95 | | | 0.74 | 1.22 | NS | | 0.96 | | 0.75 | | 1.23 | NS | | |
| Lifetime TBI | 2.80 | 1.43 | 5.49 | *** | |  | | |  |  |  | | 1.94 | | 1.02 | | 3.70 | * | | |
| Hazardous Drinking |  |  |  |  | | 5.09 | | | 2.83 | 9.17 | *** | | 4.58 | | 2.54 | | 7.93 | *** | | |
| Suicide attempts |  |  |  |  | | 6.41 | | | 2.52 | 16.27 | *** | | 5.71 | | 2.18 | | 14.91 | *** | | |
